# Development of the generic Community Infant and Young Child Feeding Counselling Package

**DOI:** 10.1111/mcn.12946

**Published:** 2023-03-29

**Authors:** Christiane Rudert, Peggy Koniz‐Booher, Mary Lung'aho, Maryanne Stone‐Jimenez, Maaike Arts, France Bégin

**Affiliations:** ^1^ Nutrition Section UNICEF East Asia & Pacific Regional Office Bangkok Thailand; ^2^ The Strengthening Partnerships, Results, and Innovations in Nutrition Globally (SPRING) Project John Snow, Inc. (JSI) Boston Massachusetts USA; ^3^ Independent Consultant USA; ^4^ Nutrition Section, Programme Division UNICEF New York New York City New York USA

**Keywords:** breastfeeding, breastfeeding support, child feeding, community‐based, counselling, infant and child nutrition

## Abstract

Infant and young child feeding (IYCF) promotion is a key component in the set of high impact interventions to improve nutrition. The literature provides evidence of the positive impact of IYCF promotion through various platforms, including communities. In 2009, UNICEF and WHO agreed that a global, “generic” IYCF package of resources and tools to plan, implement, and monitor community‐based IYCF programmes and to build skills of community‐based workers was needed. In 2010, the UNICEF Community Infant and Young Child Feeding Counselling Package was finalized and field tested under a strategic collaboration between UNICEF New York and Nutrition Policy and Practice and the Center for Human Services/University Research Company. The Package includes 11 tools to guide adaptation of the materials, the design, planning, and implementation of IYCF programmes and the training, monitoring, supervision, and mentoring of community workers, using an interactive and experiential adult learning approach. The Package was rolled out from 2011 onwards and by 2017 was implemented in 87 countries. In 2013, UNICEF and the United States Agency for International Development‐funded Strengthening Partnerships, Results, and Innovations in Nutrition Globally project started planning the evaluation, and a study site was selected in Nigeria to assess the efficacy and effectiveness of the Package on IYCF practices, knowledge, and worker skills. This article describes the need for and development of the Package, its content and approach to skills building, as well as its current implementation. Finally, it makes the case for the evaluation of the Package, which is covered in the other papers in the Supplement in relation to the Nigeria evaluation.

**Key messages**

- The generic Community Infant and Young Child Feeding Counseling Package was developed in 2010 to address a global need for a set of counseling tools for community workers
- The Package includes 11 tools to guide adaptation of the materials, the design, planning, and implementation of IYCF programmes, and the training, monitoring, supervision and mentoring of community workers.
- The Package was rolled out from 2011 onwards and by 2017 was implemented in 87 countries.

## 1 INTRODUCTION

The objective of this article is to provide the background and rationale for development of the generic UNICEF Community Infant and Young Child Feeding (C‐IYCF) Counselling Package to describe its content and approach to skills building and a picture of the status of its current implementation globally. It presents evidence to support the prioritization of particular practices as well as the approaches to promoting them that are employed by the Package. Finally, it makes the case for the evaluation of the Package, the methodology and results of which are covered in the other papers of the Supplement.

The methodology for the compilation of the information presented in this article includes a review of the scientific literature to present key evidence of the importance of IYCF promotion, which is not intended to be exhaustive; searches of the websites of key organizations working on IYCF, such as UNICEF, the World Health Organization (WHO), and the United States Agency for International Development (USAID), for IYCF case studies and implementation tools and materials; and analysis of the IYCF module of the UNICEF NutriDash online information platform; and a significant portion of the information is derived from the personal experience and documentation of the team involved in the development and implementation of the Package since its inception.

Infant and young child feeding (IYCF) counselling, education, and support, collectively referred to as “IYCF promotion,” is a key component in the set of high impact, evidence‐based interventions for nutrition. The impact has been assessed through the various contacts and channels in which IYCF promotion may take place, including various health facility contacts along the continuum of care, in the community, at home, or through mass and social media. The summary of evidence in *The Lancet* 2013 Nutrition Series (Bhutta et al., 2013) showed significant effects of IYCF promotion on improving breastfeeding practices, including that it increased exclusive breastfeeding by 43% at Day 1 and by 90% from 1 to 5 months. Combined individual counselling and group education and support seemed to have more impact than individual counselling or group sessions alone. The meta‐analysis of interventions to improve breastfeeding practices in *The Lancet* Breastfeeding Series 2016 reaffirmed the significant effects of IYCF promotion and noted that community‐based interventions, including individual counselling, group education, and social mobilization, with or without mass media, were similarly effective, increasing timely breastfeeding initiation by 86% and exclusive breastfeeding by 20% (Rollins et al., 2016). In terms of improving complementary feeding practices among children aged 6–23 months, as measured by minimum meal frequency, dietary diversity, and acceptable diet, the 2013 Lancet Series also found significant impacts of nutrition education on height and weight gain and height for age (HAZ) scores to assess stunting levels in food secure populations and HAZ and weight for height (wasting) in food insecure populations.

The WHO‐UNICEF Global Strategy for Infant and Young Child Feeding (WHO/UNICEF, 2003) gives prominence to community IYCF promotion. Various published reports on C‐IYCF promotion from UNICEF, WHO, and USAID also highlight the importance of community‐based IYCF promotion and provide lessons on best practices and recommendations for improvements. For example, a compendium of case studies (WHO/UNICEF/USAID, 2008) emphasized that more attention needed to be given during training to interpersonal counselling skills. Similarly, the USAID report on community interventions to promote optimal breastfeeding (USAID/Carolina Breastfeeding Institute, 2012) noted that excellent trainings in counselling skills to support both promotion and skilled support for breastfeeding, coupled with follow‐up and monitoring by respected, supportive supervisors/mentors, were key components of the most successful projects. This review also emphasized the importance of adult learning techniques. It was also noted at the time that most of the publications and guidance on C‐IYCF promotion, such as the WHO *Strategy for Community‐based Breastfeeding Promotion* (WHO, 2003), focused on breastfeeding and did not place much emphasis on complementary feeding.

WHO convened a consultation on strategies for scaling up protection, promotion, and support of breastfeeding at the community level in April 2008 (Casanovas & Saadeh, 2009) during which the findings of the “Learning from large‐scale community‐based programmes to improve breastfeeding practices” case studies were presented, and strategies for expanding coverage of IYCF interventions were identified. Among the latter, the consultation agreed that strategic national plans for capacity building and sustained implementation of C‐IYCF were needed, rather than the ad hoc and project‐focused trainings commonly undertaken.

Further, it was recommended that capacity building of community health workers (CHWs) needed to focus on core competencies and how these should be acquired and sustained, with an emphasis on building interpersonal communication and counselling skills. Although the meeting focused on breastfeeding, the importance of nutrition during pregnancy and lactation was recognized, and there was an agreement that breastfeeding and complementary feeding counselling needed to be linked in order to encourage a natural continuum of relevant information and support for mothers during the child's first two years of life.

Packages for in‐service training of health facility‐level workers on IYCF had been published, including the WHO‐UNICEF Integrated IYCF Counselling Course (2006), which contains modules on breastfeeding, complementary feeding, and HIV and infant feeding, as well as the original *Breastfeeding Counselling* course (WHO, 1993). This training package assumes medical or nursing training and was not intended to be used or adapted for CHWs. It featured primarily traditional, didactic learning methods using PowerPoint presentations with some exercises to practise skills. Training on this package was conducted in around 40 countries, and some evaluations and reviews were undertaken, but comprehensive documentation and an up‐to‐date picture of its use are not currently available.

In relation to community‐based packages, WHO/UNICEF materials for training community health workers under development around the same time as the C‐IYCF Package included the package on “Caring for Newborns and Children in the Community,” composed of three blocks: (a) *Caring for the Sick Child in the Community* (WHO, 2011), (b) *Caring for the Newborn at Home* (WHO, 2015a), and (c) *Caring for the Child's Healthy Growth and Development* (WHO, 2015b). IYCF promotion was a small component of these modules but did not feature building counselling skills or any depth of content on breastfeeding and complementary feeding.

Other training packages used in multiple countries included the USAID‐sponsored Essential Nutrition Actions^1^ training package for health facility workers and also included a shorter community module. It was developed in 1997 and was implemented in 22 countries in Africa and Asia by 2010. In addition, in a number of countries, various nongovernmental organizations and governments had also developed counselling and training materials on IYCF.

Based on the evidence, experiences, and technical consultations, it was widely acknowledged that in many low‐ and middle‐income countries, C‐IYCF promotion represented a key pillar of strategic IYCF programming. However, at the time (2009), neither UNICEF nor WHO had produced and published a comprehensive package of resources and tools to plan, implement, and monitor C‐IYCF programmes and to build skills of community‐based workers in IYCF counselling.

### 1.1 Development of the generic Community IYCF Counselling Package

In 2009, a year after the WHO community‐based breastfeeding consultation, UNICEF and WHO agreed that a global, generic IYCF counselling package was needed, and UNICEF committed to lead its development. In 2010, the first edition of the “generic” or global UNICEF Community Infant and Young Child Feeding (C‐IYCF) Counselling Package (referred to as “the Package” hereafter) was finalized and field tested under a strategic collaboration between UNICEF and the combined technical and graphics design team of Nutrition Policy and Practice and the Center for Human Services, the not‐for‐profit affiliate of the University Research Company.

The Package draws from the original 2006 Essential Nutrition Actions training package (latest edition Guyon, Quinn, Nielsen, & Stone‐Jimenez, 2015), the WHO Breastfeeding Counselling Course (1993), the integrated IYCF counselling package (WHO/UNICEF, 2006) and other materials used by Care and the University Research Company/Center for Human Services in East Africa. It covers IYCF practices, maternal nutrition, hygiene and sanitation, feeding the sick child, infant feeding and HIV, recognizing danger signs, and family planning. It underwent a wide peer review process involving expert institutions, including WHO, and individuals. The first edition was published at the end of 2010, and in 2012, the Package was revised and updated after a number of field tests, regional trainings and country‐level experiences. At that time, UNICEF also added a supportive supervision/mentoring and monitoring module to the Package and incorporated selected information regarding early childhood development from the WHO *Care for Child Development* module (WHO, 2012). UNICEF also published a complementary Infant and Young Child Programming Guide on all aspects of IYCF including community based counseling for programme planners (UNICEF, 2011).

### 1.2 Contents of the Package

**Figure 1:**
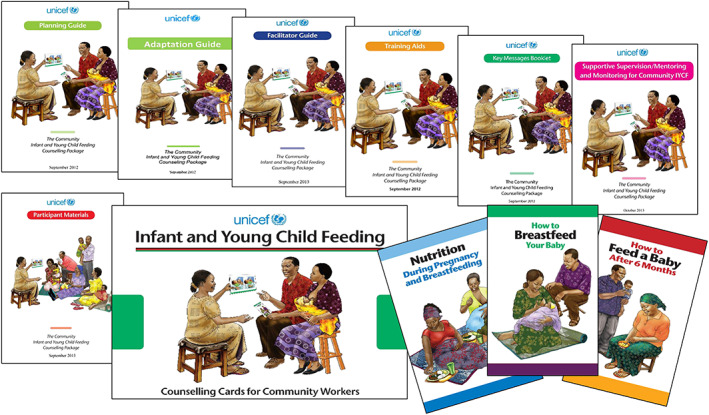
Components of the generic Community IYCF Counselling Package

The Package includes a total of 11 tools to guide the design, planning, and implementation of IYCF programmes and the training, monitoring, supervision, and mentoring of community workers (CWs), both paid CHWs and unpaid volunteers. The training tools aim to equip community workers, using an interactive and experiential adult learning approach, with relevant knowledge and skills on the recommended breastfeeding and complementary feeding practices for children from 0 to 23 months. The training focuses on counselling, problem solving, negotiation, communication skills, and the effective use of the counselling tools and related job aids for IYCF promotion in the community. The illustrations in the counselling materials and training aids were designed with the photo‐to‐illustration technique. They are of high quality, clear, and technically correct (Figure 1).

The Package is comprised of the following 11 tools:

1. The Planning Guide outlines a series of steps and includes key points about the systems and structures needed to make IYCF counselling in the community function optimally and in a sustained way as part of a broader IYCF, nutrition, or health programme. It emphasizes that assessment of existing community structures, programmes and policies, financing, community groups and partners, and support systems should always precede the planning and implementation of C‐IYCF interventions, including trainings.
2. The Adaptation Guide provides a number of specific tools, or job aids, for national or local stakeholders to use in order to adapt the Package for their setting. The Adaptation Guide recognizes that each country or setting potentially interested in using the Package has unique sociocultural specificities, including dietary behaviours, clothing styles, and linguistic characteristics, that need to be taken into consideration and ultimately reflected in the training content and communication materials. This adaptation guide is a unique feature of the generic Package and may have been a factor in its adoption in a large number of countries, given that it provides a detailed framework on how to contextualize global best practice.
3. The Facilitator Guide is for trainers or facilitators to train CWs in technical knowledge related to key IYCF practices, essential counselling skills, the effective use of counselling tools and other job aids and the development of Action Plans for Community IYCF programming post‐training.
4. The Participant Materials is for CWs to use during and after the training. It includes technical information presented during the training (“handouts” from the Facilitator Guide), tools for assessment of IYCF 3‐Step Counselling (“assess, analyse, and act”), as well as supportive supervision activities.
5. The 24 IYCF Counselling Cards are illustrations of key IYCF concepts and behaviours for CWs to use with mothers, fathers, and other caregivers. CWs are encouraged to select topics and the corresponding counselling cards, based on priorities identified during each counselling session, support group, or action‐oriented group talks. In the generic Package, there is no text written on the back of the counselling cards (which is different from many others), because CWs with a low level of literacy are trained in using the illustrations to guide the discussion. The aim of the cards is to trigger dialogue and not a one‐way didactic presentation of information, which often happens when messages are simply read out loud from a card.
6. The Key Messages Booklet consists of the specific messages related to each of the IYCF Counselling Cards and also includes copies of the three Take‐home Brochures and was created as a personal job aid for the CW.
7. Three Take‐home Brochures were designed to complement the counselling card messages and are used as individual reminder aids given to mothers, fathers, and other caregivers about key maternal nutrition, breastfeeding, and complementary feeding practices.
8. Training Aids were designed to complement the training sessions by providing visuals to help Participants better grasp and retain technical knowledge and concepts.
9. Supportive Supervision/Mentoring and Monitoring is a one‐day training module that aims to build skills of supervisors to mentor and monitor performance of CWs both in terms of the quality and coverage of activities and to help strengthen performance where gaps are identified. The module includes a menu of tools and checklists for monitoring, supportive supervision, quality assessment, and mentoring. This module also introduces the potential of monitoring the coverage of counselling among pregnant women and caregivers of children under two at community level and includes tools to do so.
10. An Orientation Presentation on the Package provides an overview of the Package and the training approach for various levels (national, subnational, and district), and partners as part of the process of introducing the Package
11. Compendium of “Clip Art” was initially developed for use by national or local stakeholders in adapting the Package for use in their own settings. In 2017, UNICEF and the USAID‐funded Strengthening Partnerships, Results, and Innovations in Nutrition Globally (SPRING) project collaborated on the development of a digital image bank^2^ to host the high‐quality illustrations developed for the generic Package and for several country‐specific adaptations. The image bank provides easy access to a wide variety of IYCF images and includes guidance on the photo‐to‐illustration process that can be used to adapt the illustrations or create new ones.

### 1.3 Skill‐building focus in the training component of the Package

The competency skills of CWs included in the Package are the following: counselling skills, including those required for basic individual counselling (listening and learning, building confidence, and giving support), and IYCF 3‐Step Counselling/reaching‐an‐agreement; problem‐solving and negotiation; breastfeeding and complementary feeding skills; communication skills and facilitation of mother‐to‐mother support groups and action‐oriented group session facilitation; and supportive supervision and monitoring skills.

The training component of the Package is intended to equip CWs with technical knowledge of recommended IYCF practices and basic counselling, negotiation, and communication skills to support caregivers to adopt and sustain these practices. Emphasis is placed on building skills for one‐on‐one counselling (listening, problem solving, and negotiating) and on facilitating groups (support groups and action‐oriented groups). The C‐IYCF training aims to build CWs' knowledge and skills. Therefore, CWs are expected to practise the skills throughout the training. A participant who has mastered the IYCF knowledge and skills will, with experience, build competence to help caregivers. If a participant can apply a skill correctly, then it is assumed that he or she has the relevant knowledge.

For national Master Trainers of Trainers, who oversee the cascade training of health workers and others who train the CWs, the following training competencies are added: planning a training/learning event; using learning outcomes to develop objectives; putting principles of adult learning into practice; developing tools for monitoring and evaluation of learning activities; monitoring and evaluating learning activities; and conducting a supportive supervisory visit.

One‐on‐one counselling can be broken down into three steps: assessment, analysis, and action. During the C‐IYCF training, participants build their skills in each—determining the situation and needs of caregiver and child, identifying and prioritizing any difficulties that the caregiver may have, determining what limited and age‐appropriate information or suggestions may best meet the needs of the caregiver, and developing a discussion with them—that is, talking and negotiating with the caregiver about trying a small “do‐able action” to address her particular situation rather than just passing along “messages” or “advice.” For the counsellor and mother of a child aged 6–23 months to agree on a suitable do‐able action, the counsellor needs to be able to assess age‐appropriate feeding frequency; amount, texture, and variety of food; active or responsive feeding; and hygiene. Then the counsellor needs to analyse any deviations and prioritize the important deviations. Also, the counsellor needs to explore any issues or barriers that may be preventing achievement of one of the critical factors and together with the caregiver, problem‐solve to arrive at a workable solution (barriers may be knowledge, culture, social, and/or material). Follow‐up with the caregiver is important to get complementary feeding back on track (step‐by‐step); link caregiver with community/group support and other supportive programmes that can help to monitor or keep an eye on child's health, nutrition, and growth.

The training materials also seek to build CWs' skills in facilitating groups (support groups and action‐oriented group education sessions). Traditionally, group or “educational” talks are organized to communicate ideas or convey information to a group. Usually, a leader directs the group talk, and group participants ask and answer questions. In a group session, CWs personalize the information by observing, thinking, trying, and acting. At the end of each session, participants are asked to try a new or different behaviour (a “small do‐able action”). In this way, the session becomes an opportunity for the CW and the participants to learn about the barriers they are facing or anticipate facing in adopting optimal IYCF practices, share their experiences, discuss possible solutions, and provide mutual support. To do this, CWs learn about group dynamics and are trained in group facilitation techniques.

### 1.4 Technical content

The technical content of the Package is built around the international recommendations for optimal feeding of infants and young children from birth through the first 2 years of life (WHO, 2003). The Package is intended to identify gaps between actual and recommended infant and young child feeding practices in communities; raise awareness among health workers on the importance of the recommended feeding practices for children from 0 to 23 months; sensitize health personnel and CWs about key contact points within health programmes for meeting with mothers, fathers, or other caregivers to discuss and support recommended IYCF practices; and increase the knowledge of CWs in order to enable them to help caregivers to adopt and sustain priority IYCF practices.

Complementary feeding receives significant emphasis and detailed coverage in the Package, recognizing that many previous community‐based programmes and trainings had a greater focus on breastfeeding counselling and acknowledging that complementary feeding behaviours and practices are complex and frequently difficult to improve.

### 1.5 Training approach

The Package uses a competency‐based, participatory approach that uses adult learning principles and is tailored to participants with limited literacy. The practical field component reinforces theory learned in the classroom and gives participants the opportunity to develop the practical skills. Participants also act as observers and resource persons for each other. Sharing of experiences is encouraged throughout, recognizing the widely acknowledged theory that adults learn best by reflecting on their own personal experiences. The interactive approach of the Package is based on the learning theories developed by Paulo Freire (listening‐dialogue‐action) and Jane Vella (Dialogue Education; Vella, 2002). “Problem‐posing education” affirms men and women as beings in the process of becoming (Freire, 1968).

The Package suggests that in order to train a cadre of CWs, there must first be a cadre of master trainers capable of training CWs who often have a low level of education or literacy. The approach to doing this differs from some other cascade training approaches in that the master trainers are trained using the same adult learning methodology, principles, and materials as they will use when they train CWs—that is, the training is “modelled” so that the trainers have both seen and experienced the training as they will deliver it. A separate training of trainers curriculum is not used for implementing the Package. The principle behind this is that it is not realistic to train the trainers differently, using PowerPoint presentations in some instances, and then expect that they will be able to train using a different training approach with different training materials.

The papers on the scaling up, barriers, and enablers and the impact evaluation of the Package in Nigeria describe how these various elements of the design and intent for the implementation of the Package actually panned out on the ground.

### 1.6 Roll‐out and monitoring of the Package

In late 2010, the Package was finalized and disseminated to all UNICEF regional and country offices. It was also introduced and promoted during UNICEF nutrition network meetings, country visits, and other opportunities. In 2011, a detailed strategy and plan was developed to conduct training sessions on the Package, from which regional master trainers would be selected.

Creating a critical mass of professionals at the regional level, with involvement of regional centres of excellence and expert facilitators, was an important step in the roll‐out plans. Having a pool of regionally based master trainers expanded the availability of trainers for the Package and reduced the dependence on global consultants. Teams of master trainers were trained in 2011, and they then led the training of trainers process in countries. A profile for trainers was developed so that countries and regions could select the most appropriate people to help ensure good quality.

By the end of 2011, around 20 countries had reported to UNICEF that they had already reached an advanced stage of local adaptation or initiated capacity development, compared with those considering or beginning to plan for the introduction of the Package. A list of a further 20 target countries for roll‐out in 2012 was developed, based mainly on expressions of interest from countries without existing national community IYCF packages and implementation and with poor IYCF practices identified in national surveys and also including others identified by UNICEF according to those two criteria.

Monitoring of the adaptation and implementation of the Package was initially undertaken through an annual questionnaire (2011–2012) sent by UNICEF to its country offices using the Package. The questionnaire included the status of materials and main adaptations made; the main coverage and performance indicators monitored; scale—in how many districts out of total in the country is the Package being implemented; number of community workers trained (out of total in country, if known); total cost to roll out training and implementation to date; main partners; constraints and challenges; and plans for the following year. From 2013 onwards, the monitoring of progress on the implementation of the Package was undertaken through the annual NutriDash^3^ exercise. (UNICEF, 2013). NutriDash is a web‐based data platform completed by UNICEF's country offices on an annual basis. It includes several modules, including one on IYCF. It is currently mostly for internal UNICEF use and selected partners for selected interventions.

**Figure 2:**
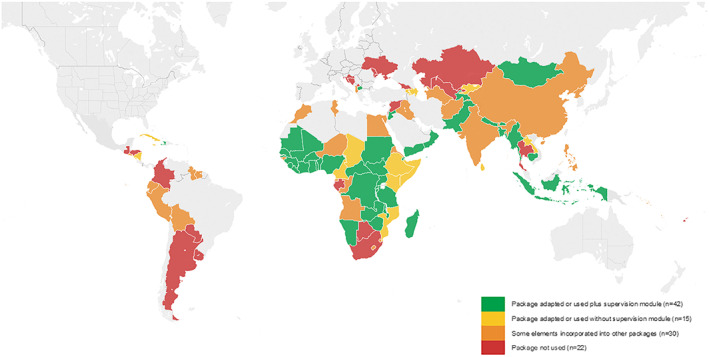
Uptake of the generic C‐IYCF Package in 87 countries (Source: UNICEF NutriDash database 2017)

In 2017, of the 109 countries that completed the UNICEF NutriDash module on IYCF (Figure 2), a high proportion—a total of 87 countries or 80%—reported using all or some elements of the Package: 42 reported using the entire Package plus the supervision module, 15 reported using the Package without the supervision module, and 30 reported using parts of the Package in another package. Of the responding countries, 22 reported not using the Package at all (UNICEF, 2017). It is noted that this 20% of UNICEF offices that did not use the Package are offices not supporting this component in their country programmes or represent countries already using other national IYCF materials. Those which adopted only parts of the Package already had other materials and used the Package to enhance those materials.

### 1.7 Next steps for the Package at global level

The next steps for the content and implementation of the Package include the following: (a) Review the implementation guidance and technical content of the Package in light of the findings of the evaluation conducted in Nigeria and other impact evaluations. (b) Produce a process review of its implementation globally, with selected country case studies, reflections on lessons learned, the extent to which the adult learning techniques were applied in the training approach, the interactive counselling and group sessions were used by CWs and the supervision, mentoring, and monitoring tools were used. It will also review the scale, quality, and sustainability of counselling where possible. (c) Update the modules of the Package as applicable, based on the evaluations and the above process review and lessons learned. (d) Update the content to enhance the early childhood development aspect of the Package so that it can serve as a single resource for IYCF and Care for Child Development in both frontline health facilities and at community level. This next edition will also include updates to give more focus to the prevention of overweight and limiting the use of unhealthy foods, snacks, and drinks. The overweight prevention aspect was not given significant attention in the previous versions, but given rapidly rising rates of child overweight in many countries and the greater focus on overweight prevention by Governments and UNICEF, it is considered important to enhance this aspect. And (e) in contexts where Integrated Community Case Management and/or Community Management of Acute Malnutrition are implemented, pursue greater alignment of these with the C‐IYCF Package implementation.

### 1.8 Rationale for the evaluation of the Package

Although the importance of conducting a formal evaluation of the Package to assess its impact was recognized, securing the necessary funding was difficult. Finally, in 2013, SPRING and UNICEF initiated planning for a formal evaluation of the Package to assess the effectiveness of its impact on maternal, infant, and young child nutrition practices, caregiver knowledge and attitudes, and the skills of community workers in areas where the entire Package has been introduced “at scale.” A further aim of the evaluation was to investigate factors of the enabling environment that influenced the implementation and sustainability of C‐IYCF programming in a country where it has been introduced.

Nigeria was the country identified for this evaluation because of the high interest in and early commitment to the adaptation and adoption of the Package (Nigeria was one of the first countries to adapt the C‐IYCF Counselling Package). Nigeria also already had several years of programmatic experience with the implementation of the Package. Further, there was strong national interest from the government and partners in formally evaluating the impact of the Package in order to guide future programming decisions. UNICEF and two USAID‐funded projects—the Infant and Young Child Nutrition Project and subsequently SPRING—were also both actively supporting the C‐IYCF programme in Nigeria.

### 1.9 Contents of this supplement

In additional to this paper on the development of the Package, this supplement contains three additional papers focused on the design of this mixed methods evaluation (Lamstein, Pérez‐Escamilla, Koniz‐Booher, Adeyemi et al., 2020), the environmental factors enabling and limiting the success of the Package (Pérez‐Escamilla, 2020), and the impact of the Package on breastfeeding practices (Lamstein, Pérez‐Escamilla, Koniz‐Booher, Stammer, et al., 2020). In addition, the commentary reflects on the issues of scalability of the Package and the importance of engaging local leaders, and two case studies from Rwanda and Indonesia highlight the experiences of implementing the Package in different contexts.

## CONFLICTS OF INTEREST

The authors declare they have no conflicts of interest.

## CONTRIBUTIONS

CR wrote the paper. PKB, ML, MSJ, MA, and FB reviewed and made contributions to the drafts of the paper. All authors have read and approved the final manuscript.

## Footnotes

1
https://www.slideshare.net/COREGroup1/quinn-ena-1 (2010)

2
https://iycf.spring-nutrition.org/

3 NutriDash is a global nutrition programme monitoring platform that collects annual data from UNICEF country offices on national implementation of IYCF, multiple micronutrient powders, salt iodization, Vitamin A supplementation, and the treatment of severe acute malnutrition. The most recent data available is from 2017.
